# Supplementary material for: FERN – a Java framework for stochastic simulation and evaluation of reaction networks
Source: BMC Bioinformatics. 2008 Aug 29;9:356. doi: 10.1186/1471-2105-9-356 (PMC2553347; doi:10.1186/1471-2105-9-356)
Supplement: Additional file 1 — FERN distribution, Version 1.3. This archive contains the FERN source code and binaries as well as documentation and example models in FernML and SBML. [file 1471-2105-9-356-S1.zip › fern/doc/javadoc/fern/simulation/observer/FireTypeObserver.html]

FireTypeObserver


---


|  |  |  |
| --- | --- | --- |
| **Overview** \| **Package** \| **Class** \| **Use** \| **Tree** \| **Deprecated** \| **Index** \| **Help** | |  |
| **PREV CLASS**   **NEXT CLASS** | **FRAMES**    **NO FRAMES**     **All Classes** |  |
| SUMMARY: NESTED \| FIELD \| CONSTR \| METHOD | DETAIL: FIELD \| CONSTR \| METHOD |  |


---


## fern.simulation.observer Class FireTypeObserver

```
java.lang.Object
  fern.simulation.observer.Observer
      fern.simulation.observer.FireTypeObserver
```

**All Implemented Interfaces:**: GnuPlotObserver

---

``` public class FireTypeObserver extends Observer implements GnuPlotObserver ```

Observes the different types of firings.

If the simulation is repeated, the average numbers are calculated.

**Author:**
:   Florian Erhard

**See Also:**: `Simulator.FireType`

---

**Constructor Summary**

`FireTypeObserver(Simulator sim)`
  
          Create the observer for the given simulator.


| **Method Summary** | |
| --- | --- |
| `void` | `activateReaction(int mu, double tau, Simulator.FireType fireType, int times)`             Counts the firing for the given `Simulator.FireType` |
| `void` | `finished()`             Calculates the average over all runs of the simulator. |
| `String[]` | `getStyles()`             Gets the styles for the columns. |
| `void` | `started()`             Do nothing. |
| `void` | `step()`             Do nothing. |
| `void` | `theta(double theta)`             Do nothing. |
| `GnuPlot` | `toGnuplot()`             Creates a new `GnuPlot` object and passes the actual observer data to it. |
| `GnuPlot` | `toGnuplot(GnuPlot gnuplot)`             Passes the actual observer data to a `GnuPlot` object. |
| `String` | `toString()` |

**Methods inherited from class fern.simulation.observer.Observer**

`applyLabelFormat, getLabel, getLabelFormat, getNumSimulations, getPrintWriter, getSimulator, getTheta, getTitlesCommand, print, setLabelFormat, setPrintWriter, setSimulator, setTheta`

**Methods inherited from class java.lang.Object**

`clone, equals, finalize, getClass, hashCode, notify, notifyAll, wait, wait, wait`

**Constructor Detail**

### FireTypeObserver

```
public FireTypeObserver(Simulator sim)
```

:   Create the observer for the given simulator.

    **Parameters:**: `sim` - simulator


**Method Detail**

### activateReaction

```
public void activateReaction(int mu,
                             double tau,
                             Simulator.FireType fireType,
                             int times)
```

:   Counts the firing for the given `Simulator.FireType`

    :   **Specified by:**: `activateReaction` in class `Observer`
    :   **Parameters:**: `mu` - the reaction which is supposed to fire: `tau` - the time the reaction fires (at this time `Simulator.getTime()` does not necessarily yield the firing time): `fireType` - the type of the firing: `times` - TODO

---


### finished

```
public void finished()
```

:   Calculates the average over all runs of the simulator.

    :   **Specified by:**: `finished` in class `Observer`

---


### started

```
public void started()
```

:   Do nothing.

    :   **Specified by:**: `started` in class `Observer`

---


### step

```
public void step()
```

:   Do nothing.

    :   **Specified by:**: `step` in class `Observer`

---


### toString

```
public String toString()
```

:   **Overrides:**: `toString` in class `Object`

---


### theta

```
public void theta(double theta)
```

:   Do nothing.

    :   **Specified by:**: `theta` in class `Observer`
    :   **Parameters:**: `theta` - moment in time

---


### toGnuplot

```
public GnuPlot toGnuplot()
```

:   **Description copied from interface: `GnuPlotObserver`**
:   Creates a new `GnuPlot` object and passes the actual observer data to it.

    :   **Specified by:**: `toGnuplot` in interface `GnuPlotObserver`
    :   **Returns:**: the created `GnuPlot` object

---


### toGnuplot

```
public GnuPlot toGnuplot(GnuPlot gnuplot)
```

:   **Description copied from interface: `GnuPlotObserver`**
:   Passes the actual observer data to a `GnuPlot` object.

    :   **Specified by:**: `toGnuplot` in interface `GnuPlotObserver`
    :   **Parameters:**: `gnuplot` - the `GnuPlot` object to pass the data to **Returns:**: the `GnuPlot` object

---


### getStyles

```
public String[] getStyles()
```

:   **Description copied from interface: `GnuPlotObserver`**
:   Gets the styles for the columns. If you don't want styles, just return null!

    :   **Specified by:**: `getStyles` in interface `GnuPlotObserver`
    :   **Returns:**: styles for the columns


---


|  |  |  |
| --- | --- | --- |
| **Overview** \| **Package** \| **Class** \| **Use** \| **Tree** \| **Deprecated** \| **Index** \| **Help** | |  |
| **PREV CLASS**   **NEXT CLASS** | **FRAMES**    **NO FRAMES**     **All Classes** |  |
| SUMMARY: NESTED \| FIELD \| CONSTR \| METHOD | DETAIL: FIELD \| CONSTR \| METHOD |  |


---
